# Skin Barrier Restoration by Waste-Derived Multifunctional Adhesive Hydrogel Based on Tannin-Modified Chitosan

**DOI:** 10.1021/acsami.5c03066

**Published:** 2025-05-19

**Authors:** Martina Ferri, Francesco Ganzerli, Alberto Portone, Tiziana Petrachi, Elena Veronesi, Davide Morselli, Micaela Degli Esposti, Paola Fabbri

**Affiliations:** † Department of Civil, Chemical, Environmental and Materials Engineering (DICAM), 9296 Università di Bologna, Via Terracini 28, Bologna 40131, Italy; ‡ National Interuniversity Consortium of Materials Science and Technology (INSTM), Via Giusti 9, Firenze 50121, Italy; § Tecnopolo Mario Veronesi, Via 29 Maggio 6, Mirandola, Modena 41037, Italy

**Keywords:** chitosan, tannins, polyphenols, active wound dressings, skin barrier restoration, waste valorization

## Abstract

The development of multifunctional materials that actively enhance the wound healing process is critically important in addressing clinical and public healthcare challenges. Here, we report a multifunctional hydrogel obtained through physical cross-linking of chitosan and wood tannins for active wound management. Tannins, as polyphenolic wood-waste derivatives, act both as multifunctional additives and cross-linking agents, resulting in a stable and highly swellable hydrogel (>2000%·mg^–1^). The dressing is produced in the form of a dry and rigid film for easy transportation. After swelling, the material exhibits adequate Young’s modulus (∼7 MPa, comparable to the stratum corneum’s stiffness), improved flexibility, and suitable adhesion strength to adapt to joint movements. Polyphenolic tannins also provide the material with high antioxidant activity against DPPH radicals (100% RSA), showing potential for preventing complications during the inflammation phase. Moreover, tannins can completely block skin-damaging UV light without significantly altering the material’s transparency, thus allowing constant visual wound monitoring. Wound healing investigations on abdominoplasty-derived skin demonstrated that tannins enhance the normal skin barrier restoration process, thereby facilitating the transition toward wound regeneration. This work offers a sustainable strategy for valorizing agri-food waste in a fully biobased material to address active wound management.

## Graphical abstract

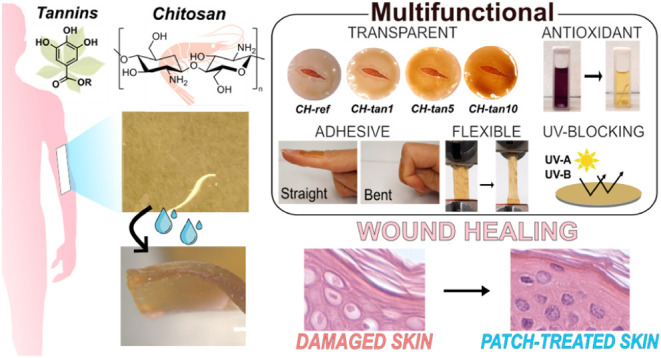

## 1 Introduction

In the past decade, hydrogels have attracted growing interest in the field of wound management thanks to their biocompatibility, high water-absorbing three-dimensional (3D) network structure, and capability of mimicking the extracellular matrix, which can significantly contribute to speeding up the healing process of the skin.^1^ At the same time, the unique mechanical properties of hydrogels, i.e., elasticity and flexibility, derived from their swelling capability, allow them to easily adapt to different wound shapes and provide comparable mechanical properties to native skin tissue.^2^ Since wound dressings are typically used for a short time and soon disposed of, hydrogels based on natural hydrophilic polymers (e.g., alginate, collagen, gelatin, dextran, hyaluronic acid, and chitosan) have gained considerable attention compared to synthetic ones.^3^ This is mainly due to their inherent biocompatibility and biodegradability, making them particularly suitable for contact with the human body and more effective in promoting environmentally friendly wound management.^4^ For example, alginate, a naturally occurring polysaccharide derived from brown seaweed, is widely used in wound dressings due to its excellent moisture-retention capability and gel-forming properties upon contact with wound exudate.^5,6^ Gelatin, a protein derived from collagen, has been highlighted for its role in chronic wound repair and is particularly indicated thanks to its biocompatibility and ability to promote cell migration.^7^ Moreover, these materials can be loaded with bioactive agents such as growth factors or nanoparticles^8,9^ to enhance tissue regeneration. Among natural hydrophilic polymers, chitosan has emerged as one of the most promising candidates for obtaining hydrogels due to its unique biological properties, including biocompatibility and antimicrobial and hemostatic properties.^10–12^

Recently, most of the research has focused on the transition from passive wound dressings, which are only used for physical wound protection, to smart multifunctional materials with synergistic effects that actively contribute to the wound healing process. This is typically achieved by incorporating active molecules/particles into the polymeric matrix, leading to biomultiactive hydrogels that can provide all-in-one advanced functions, including antimicrobial activity, adhesion, hemostasis, and anti-inflammatory, antioxidant, and UV protection properties.^13,14^

Several examples of multifunctional hydrogels, derived from the incorporation of multifunctional biobased polyphenolic additives, have been reported in the literature. Wang et al. incorporated nanoparticles of curcumin, a hydrophobic natural polyphenolic compound extracted from turmeric, into gelatin microspheres, obtaining an anti-inflammatory hydrogel dressing for diabetic wound repair.^15^ Another recent example reports the use of vitamin C, a bioactive molecule, loaded into zein/pectin formulations, resulting in an antioxidant and anti-inflammatory platform for skin wound care.^16^ Other natural polyphenolic components like tea polyphenols,^17^ anthocyanins,^18^ and some flavonoids^19,20^ have been successfully incorporated into hydrogels showing antioxidant activity, which is particularly important for diabetic wound healing applications.^21^ Apart from combating oxidative stress, the high hydroxylation degree of flavonoids can also give the hydrogel antibacterial, antifibrotic, and anti-inflammatory effects.^22^ However, most of the aforementioned additives are still obtained through lengthy extraction and purification procedures to be used as single and pure molecules. A truly sustainable upcycling strategy should prioritize minimal processing steps capable of producing bioactive mixtures suitable for immediate application in biomedical fields.

Tannins are polyphenols synthesized by plants as secondary metabolites, primarily to protect themselves against both biotic and abiotic stress factors.^23^ Among them, hydrolyzable tannins can be easily extracted using water as a complex mixture of molecules and are traditionally used in low-value applications, such as leather tanning and animal feed.^24,25^ However, in recent years, they have gained attention as biobased additives with multifunctional properties (antibacterial, antioxidant, UV-blocking, among others^25^) for use in polymer-based systems across fields such as food packaging and water remediation.^26,27^ Tannic acid, the most studied representative molecule of hydrolyzable tannins, has already been proposed for wound healing applications.^28–30^ However, the extraction and purification of tannic acid as a single purified molecule from tannins require more time-consuming and expensive procedures compared to simply using the direct botanical complex mixture. Tannins used in this work, derived from chestnut wood, have been shown to be rich in a variety of polyphenolic molecules, including castalagin, vescalagin, vescalin, castalin, gallic acid, and related compounds.^31^ These compounds share an OH-rich chemical structure, which can effectively enable them to act as H-bond-based cross-linking sites for the formation of stable hydrogel networks.^32^ Consequently, the direct use of tannins can represent an effective strategy to valorize a waste, typically used for low-value products, into high-value biomedical applications.

In this work, we propose the use of a direct plant-derived waste product, specifically tannins derived from chestnut wood, as a multifunctional and waste-valorizing additive for wound healing applications. In particular, increasing amounts of tannins (0, 1, 5, and 10 wt %) have been homogeneously introduced into chitosan, which has been selected as one of the most promising renewable polymers^33^ for developing hydrogels for wound repair. The successful incorporation of tannins into chitosan resulted in transparent and flexible polymeric films, where intermolecular interactions between chitosan and tannins enabled the formation of a supramolecular complex. This allowed us to achieve a high swelling degree, improved stretchability, and adhesion to the skin. Moreover, the addition of tannins as a powerful antioxidant additive made it possible to obtain an active material characterized by enhanced scavenging activity for limiting oxidative stress. Additionally, the numerous aromatic rings in tannin’s structure suggest their potential UV-barrier properties that protect injured skin from damaging UV radiation. Finally, *in vitro* and *ex vivo* biological evaluation of the prepared hydrogels proved that the presence of tannins can significantly promote epidermal regeneration and the reacquisition of the physiological skin barrier capability.

## 2 Results and Discussion

As summarized in Scheme 1, the solvent casting technique was employed as a fast and simple preparation method for the incorporation of tannins into chitosan. More specifically, chitosan was solubilized in an aqueous solution of formic acid at 2% v/v, filtered, and mixed with a tannin solution in the same solvent. Once the solution was homogeneous, it was cast into a Petri dish. Films with increasing tannin content (0, 1, 5, and 10 wt %, coded as CH-ref, CH-tan1, CH-tan5, and CH-tan10, respectively) were obtained with homogeneous tannin incorporation after oven drying at 37 °C, followed by a final step of 16 h in a vacuum oven (detailed procedure provided in the experimental section).

**Scheme 1:**
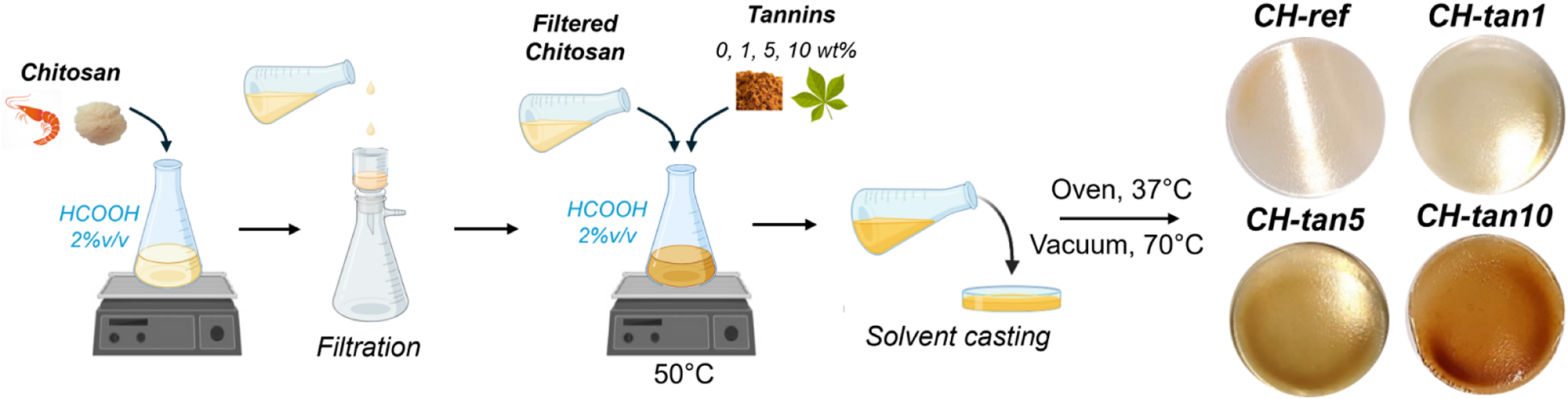
Preparation Procedure and Representative Photographs of the Obtained Films at the Investigated Tannin Concentrations (0, 1, 5, and 10 wt %)

When chitosan is combined with molecules containing numerous hydroxyl groups, several interactions can occur depending on the preparation method. If no chemical reaction occurs, the main polyphenol-polysaccharide bonding is generally governed by hydrogen bonds and minor ionic interactions.^34,35^ Fourier-transform infrared spectroscopy (FT-IR) was employed to investigate the bonding nature between the two compounds, as reported in Figure 1A. Tannin’s main bands were observed at 3400–3200 cm^–1^, 1719 cm^–1^, and 1600 cm^–1^, attributed to the O–H stretching, the carboxyl ester groups (O–C=O) stretching and the aromatic C=C stretching, respectively.^36,37^ In the CH-ref spectrum, the amide I (C=O of chitosan’s amide) and amide II band (N–H) were responsible for the bands at 1650 and 1570 cm^–1^, while the overlap of the −NH and −OH stretching vibrations was visible in the broad band at 3500–3100 cm^–1^.^38^ The latter band was subjected to a significant intensification with the increasing content of tannins in the CH-tannin films (Figure 1B), due to the increase in the polyphenolic hydroxyl groups (3400–3200 cm^–1^) of tannin’s structure, revealing the successful incorporation of the polyphenols into the polymer matrix.^37^ The formation of the hydrogel’s 3D network is probably driven by weak intermolecular interactions, likely ascribable to hydrogen bonding, occurring between the abundant hydroxyl groups of tannins and the amide and carboxyl functional groups present in the chitosan structure.^39^

**Figure 1:**
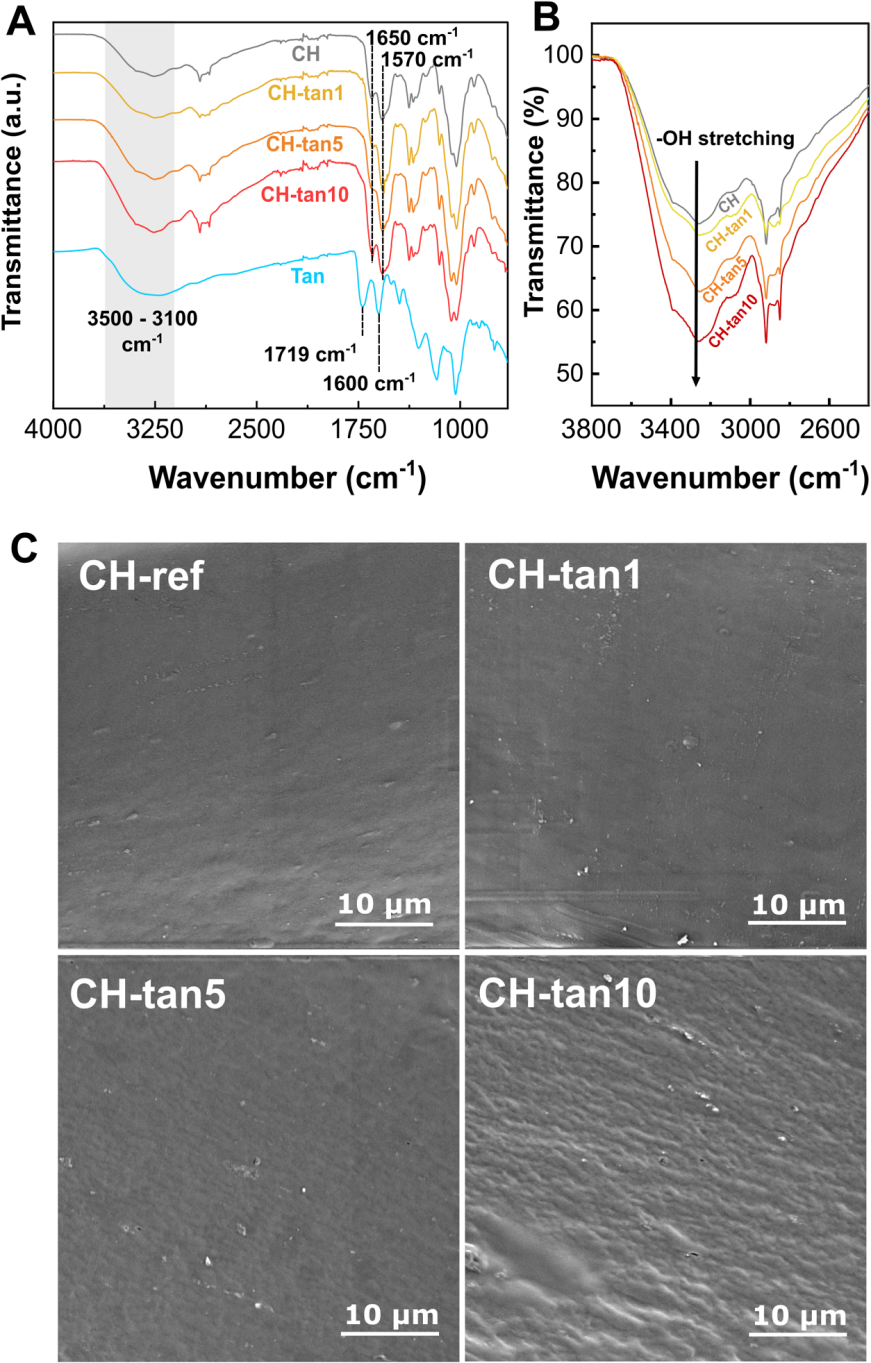
(A) FT-IR (ATR) spectra of CH-ref, CH-tannin films, and tannins (Tan). The main bands are highlighted by gray rectangle and dashed black lines. (B) Highlighted region of FT-IR spectra between 3800 and 2500 cm^–1^. Black arrow indicates the increase in the −OH broad band intensity with the tannin content. (C) SEM (secondary electrons) of CH-ref, CH-tan1, CH-tan5, and CH-tan10.

The cryofractured cross-sectional morphologies of CH-tannin films were studied through scanning electron microscopy (SEM). Figure 1C shows that tannins were well-distributed within the chitosan, as all of the morphologies presented a smooth surface. At the highest tannin concentration in CH-tan10, the material appeared to assume a slightly rough surface, which could be attributed to minor aggregation of tannins. Similar morphologies were observed in materials where tannins and chitosan were combined primarily through weak intermolecular interactions.^40,41^

The swelling behavior of a material can affect multiple aspects of the wound-healing process. First, maintaining a moist environment at the wound site is essential for absorbing wound exudates, minimizing irritation, and promoting cell migration and tissue regeneration.^42^ Moreover, the desired swollen material must display suitable flexibility to tightly adhere to the wound surface and ensure the comfort of the patient. For these reasons, the swelling degree (calculated according to eq 1) of the CH-tannin films was investigated to determine the water-swelling response of the chitosan polymer after the incorporation of an increasing content of tannins (Figure 2A). All samples began to absorb water once immersed. However, it is worth noting that the neat chitosan sample (CH-ref) exhibited poor stability, undergoing rapid and high swelling followed by disintegration in the medium, losing its structural integrity and handling capability (Figure 2B). In contrast, all formulations containing tannins demonstrated a more controlled swelling behavior, gradually reaching a swelling plateau after approximately 4 h of immersion. This suggests that an equilibrium was established between the water uptake into the material and the retroactive force applied by the tannin-chitosan interactions. These results indicate the presence of numerous interactions likely ascribable to hydrogen bonds between the numerous tannin’s −OH groups (Figure 1A,B) and the carboxyl and amide groups within chitosan chains.^43^ It is also generally expected that an increase in the additive can lead to a decrease in the swelling plateau due to a denser polymeric 3D network. In this case, this was verified when the tannin amount was increased from 1 wt % to 5 wt % (lowering from 1200 to 750%·mg^–1^). Interestingly, doubling the amount of tannins from 5 to 10 wt % did not show any further decrease in the swelling plateau value, which remained almost stable at 750%·mg^–1^. These results were further supported by the gel content and the extractable fraction,^38^ calculated according to eqs 2 and 3, shown in Figure 2C. Typically, a properly bonded macromolecular 3D network displays high gel content values and low extractable fraction values.^44,45^ Indeed, neat chitosan showed a low gel content as expected (approximately 25%), whereas it is noteworthy that when only 1 wt % of tannin was added, this value increased up to approximately 40%, and an addition of 5 wt % of tannins further increased the gel formation to almost 60%. These results suggest the formation of a denser 3D network due to the secondary interactions between tannins and chitosan macromolecules.^38^

**Figure 2:**
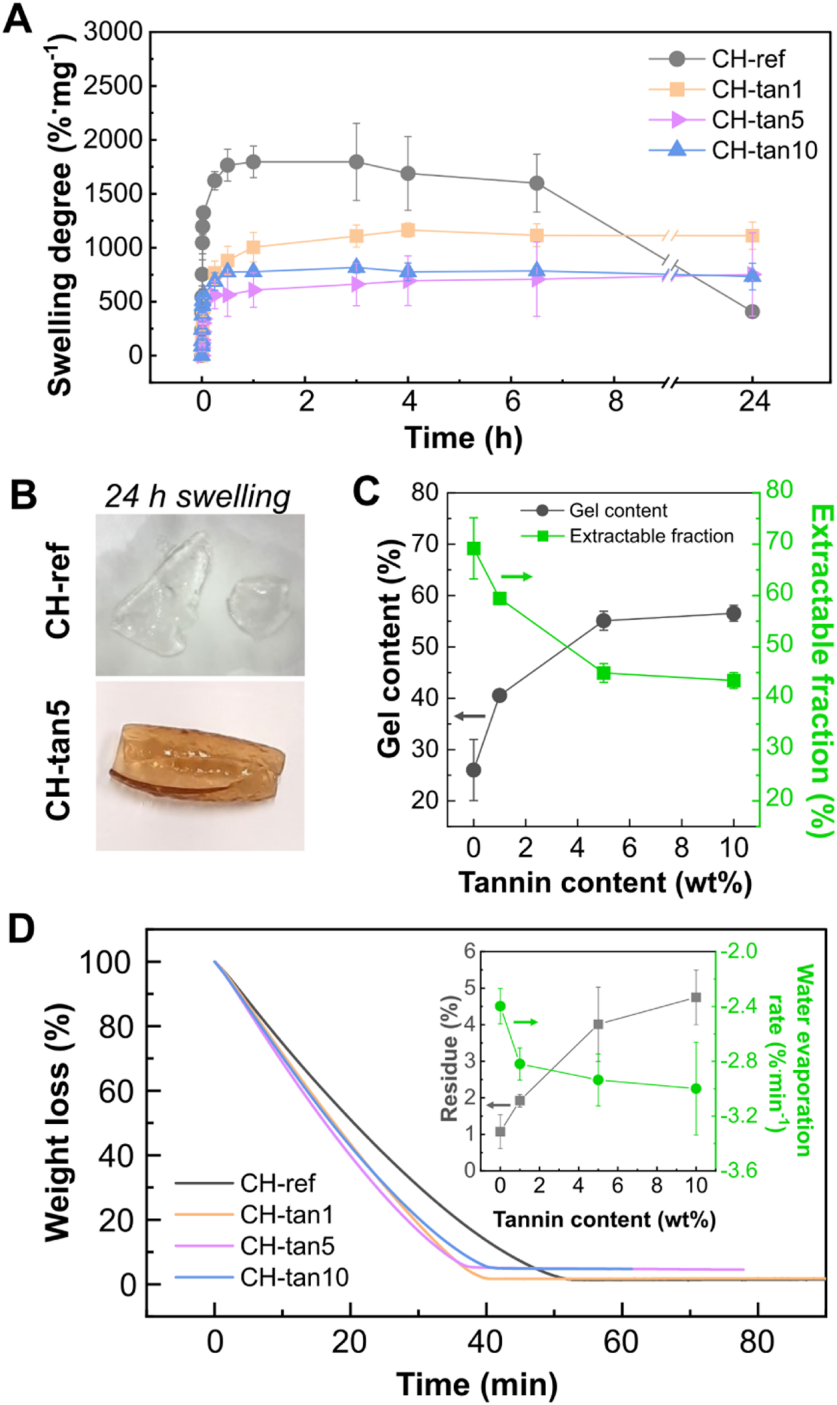
(A) Swelling degree normalized by the mass of the polymer as a function of time. (B) Representative photographs of CH-ref dissolution and its handleability loss compared to CH-tannin’s stability after 24 h of swelling. (C) Gel content (%) and extractable fraction (%) of CH-ref and CH-tannin films with increasing tannin content. (D) Weight loss (%) as a function of time for CH-ref and CH-tannin films at isothermal 37 °C. Inset shows the residue weight (%) and the water evaporation rate (%·min^–1^) as a function of tannin content in the hydrogel films.

Furthermore, the water evaporation from the fully swollen CH-tannin hydrogels was evaluated by monitoring their weight loss (%) at the isothermal body temperature of 37 °C through thermogravimetric analysis (TGA) under an airflow of 20 mL/min. Curves of water loss are presented in Figure 2D and show that all samples reached a plateau after 40 to 50 min. These results are likely driven by the forced water-loss condition induced by the experimental setup, which was selected to obtain a comparative trend of evaporation among the different formulations, where the patch is exposed to a double-sided higher airflow than in real-life practice. Tannin-containing films demonstrated a slightly higher water evaporation rate compared to the neat polymer (inset of Figure 2D). As previously reported in the literature, the less a sample absorbs water, the faster it loses its absorbed water.^38^ On the other hand, a slight increase in the residue weight (%) after drying at 37 °C was observed. The hydrophilic nature of tannins and chitosan combined with the complex polymeric network formed in the hydrogel films could be responsible for more entrapped water, which needs higher temperatures to be fully removed from the structure.^46,47^ This indicates that hydrogels with a higher tannin content are potentially more effective at retaining water and moisture at the wound site.

Besides exhibiting high swelling behavior, both mechanical resistance and skin-adhesion strength are fundamental requisites for the efficacy of wound-healing patches, ensuring easy applicability on the skin and mechanical protection from external stimuli during the healing period.^48^ The mechanical behavior of CH-tannin films was investigated through tensile tests on dry and swollen films (5 s of contact with distilled water, curves in Figure 3A). As expected, Figure 3B shows that the Young’s modulus (*E*) drops from an overall value of 2000 MPa for the dry films to approximately 7 MPa for the swollen films. Moreover, the increasing content of tannins in both dry and swollen conditions enhanced the material’s stiffness. On the other hand, there was a significant increase in the elongation at break (ε_b_) from approximately 15% for dry films to an overall value of 60% for swollen samples. This behavior is a consequence of the presence of water molecules that intercalate between the macromolecules and are free to move across the hydrogel network, resulting in a high plasticization effect.^49^ These data highlight the transition from a very rigid material in the dry state, unsuitable for skin contact but optimal for transportation and storage, to a soft, stretchable (Figure 3C), and swollen material that is more desirable for direct contact with the skin. The *E* values obtained for the swollen films were also comparable to stiffness values reported in the literature for the stratum corneum, the outermost layer of the epidermis, which range from 6 MPa for young skin to 12 MPa for aged skin,^50^ indicating their potential as skin wound dressings. Furthermore, the effect of longer swelling times (15 and 30 s) on the mechanical properties of the films was investigated. Figure S1 reveals that *E* and ε_b_ did not significantly change with varying swelling time, indicating that 5 s of swelling was sufficient to obtain a stretchable material that is easily applicable to the skin and remains well-adhered even during joint movements, as shown in Figure 3D and Video SV1.

**Figure 3:**
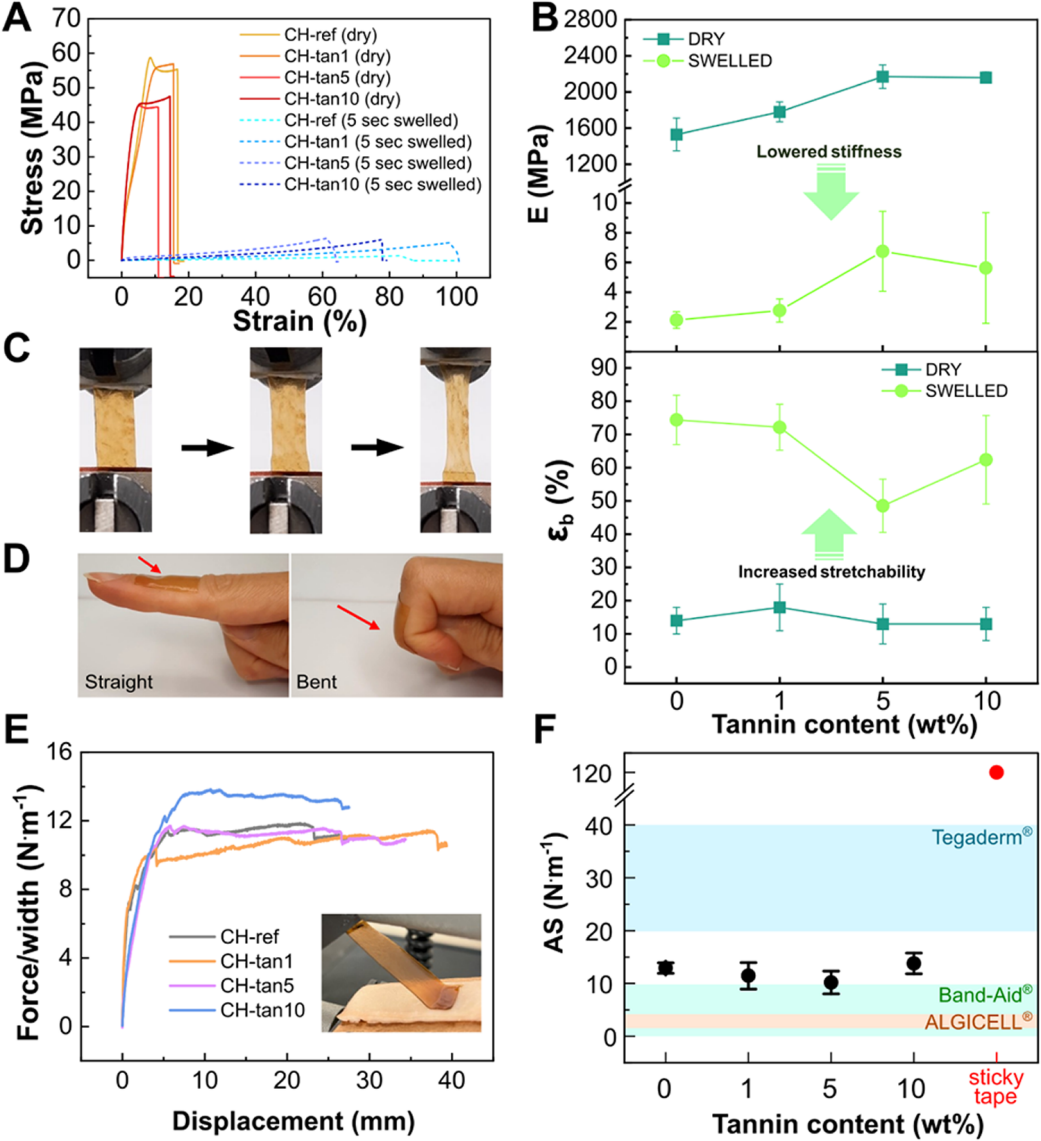
(A) Representative stress–strain curves obtained from tensile test on dry (warm colors, solid lines) and 5-s swollen samples (cold colors, dashed lines). (B) Young’s modulus (*E*, MPa) and elongation at break (*ε*_b_, %) of the dry and the 5-s swollen films. Arrows highlight the changes in the *E* and *ε*_*b*_ values. (C) Detail on the elongation of CH-tan5 swollen film during the tensile test. (D) Representative adhesion and flexibility test on the skin at the finger joint. Full video is available as Video SV1. (E) Peel adhesion (force/width) over displacement curves of the chitosan and CH-tannin films. A photograph of the test on pig skin is also reported. (F) Adhesion strength (AS) for CH-tannin samples (black dot) as a function of the tannin content and adhesion strength of sticky tape (red dot) as a comparison. Blue, green, and orange shaded areas highlight the ranges of adhesion strength of commercial dressings Tegaderm® (acrylate), BandAid® (acrylate/fabric), and ALGICELL® (alginate), respectively.^55^.

The adhesive properties of the swollen CH-tannin hydrogels were also studied using a T-peel test geometry on pig skin (the experimental measurement setup can be seen in Figure S2 and Video SV2). The curves reported in Figure 3E show that the adhesion strength (AS) remained stable at approximately 10 N·m^–1^ up to 5 wt % tannins, while a slight increase was detected at the maximum tannin content of 10 wt % (15 N·m^–1^). The polyphenolic structure of tannins is well-known to confer adhesive properties to both organic and inorganic surfaces, as it has been extensively studied for molecules with similar chemical structures, such as catechol and tannic acid.^51,52^ It is believed that, at the lower tannin concentrations of 1 and 5 wt %, the CH-tannin interactions may likely subtract numerous tannin’s −OH groups required for adhesive activity. Only when higher tannin content was introduced (10 wt %), as previously observed from the swelling behavior and gel content formation, did the CH-tannin interactions reach a threshold, leaving free hydroxyl groups that can improve adhesion due to the excess tannin.^53,54^ In Figure 3F, the AS of CH-tannin hydrogels is shown to be comparable to that of several commercial wound dressings based on various polymers, such as Tegaderm® (acrylate), BandAid® (acrylate/fabric), and ALGICELL® (alginate), as indicated by the blue, green, and orange bands, respectively.^55^ This places our material within the range of existing clinical patches in terms of adhesive performance. In contrast, the AS of a commercial sticky tape (represented by the red dot in Figure 3F) is significantly higher. However, such high adhesion is unsuitable for direct contact with wounded skin, as it may lead to detachment of newly regenerated skin and cause pain during removal. In this context, the exhibited adhesion of CH-tannin hydrogels can be considered sufficient to ensure dressing retention, while also contributing to patient comfort by ensuring painless patch removal.

It is well-known that UV light can deeply penetrate the skin, even under normal conditions, causing accelerated cell aging, skin pigmentation, and, in the worst cases, skin cancer.^56^ This phenomenon could be accentuated under damaged skin conditions. Therefore, it is highly desirable to prepare wound dressings with UV-blocking properties. Another important feature of the dressing is its transparency, which enables continuous visual inspection of the wound site without the need for removal, thus providing noninvasive monitoring for the patient.^57^

In this perspective, the optical properties of CH-tannin films were studied by collecting their UV–visible spectra as shown in Figure 4A. A clear decrease in transmittance in the UV–A and UV–B ranges was detected as the tannin content increased, resulting in better UV-blocking activity, calculated according to eq 4. Nevertheless, it is worth noting that transparency, evaluated as the transmittance at 600 nm T_600_, was not significantly affected. The photographs shown in the inset of Figure 4A prove that even at the maximum tannin-loaded film (10 wt %), the transparency was maintained at over 60%, despite the color change. As shown in Figure 4B, CH-tan5 represented the best compromise between transparency and UV-blocking activity, as it still displayed 80% of T_600_, placing it in the range of transparent materials,^58^ while ensuring an extremely high UV-blocking activity of 100% for UV–B and 99% for UV–A. These features make CH-tan5 a suitable dressing that offers both high protection against UV irradiation and effective visual monitoring of the wound.

**Figure 4:**
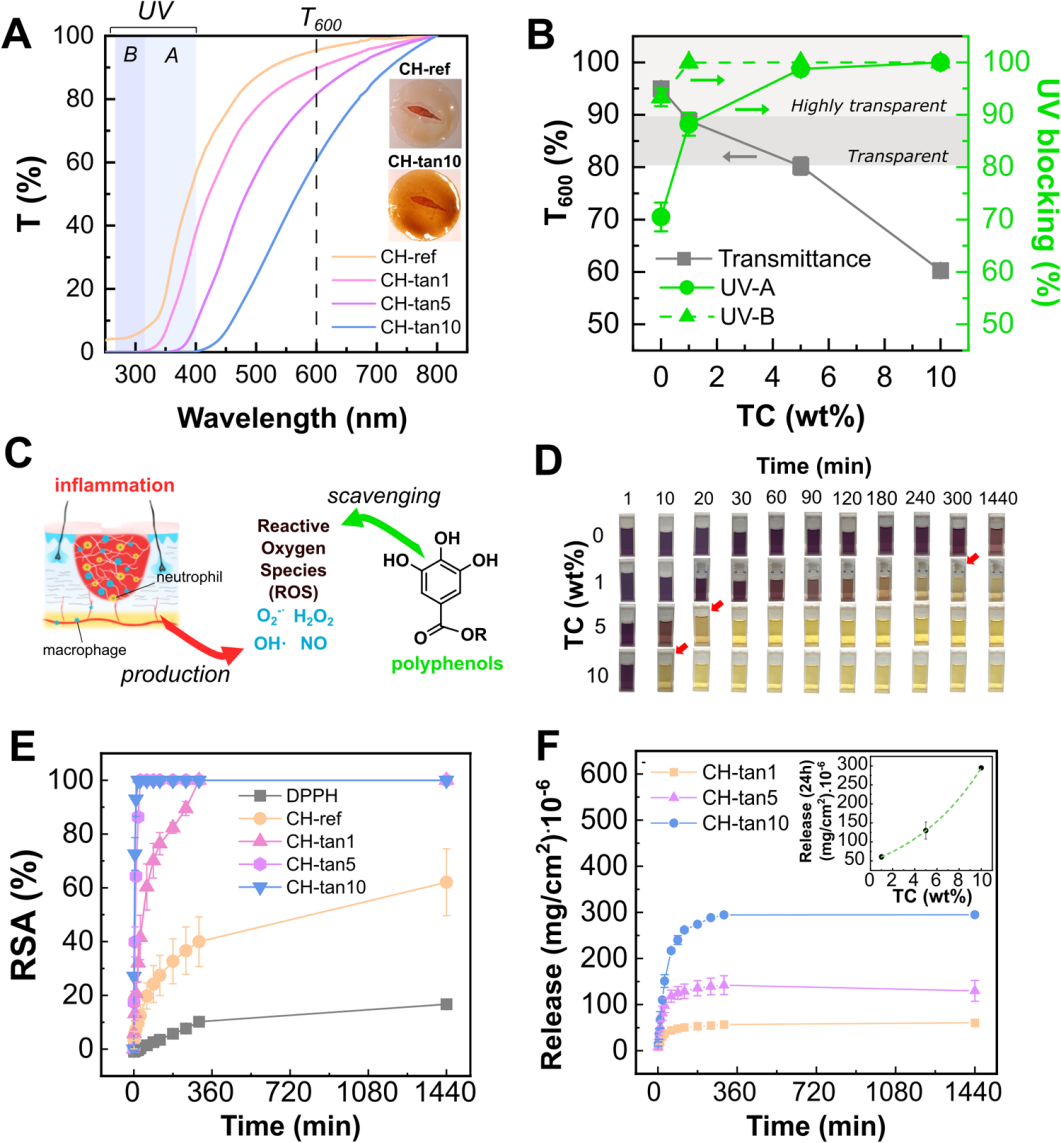
(A) Transmittance of CH-ref and CH-tannin hydrogel films in the UV–vis range. T_600_, UV–B, and UV–A ranges are highlighted. Inset shows the final transparent appearance of CH-ref and CH-tan10 on a digital wound icon. (B) Transmittance and UV-blocking properties of CH-ref and CH-tannin films. UV–A- and UV–B-blocking activity were calculated by eq 4. (C) Scheme of the effect of polyphenolic antioxidants on the reactive oxygen species (ROS) produced during the inflammatory stage. (D) Color change of the DPPH^•^ solution after the introduction of film samples of CH-ref (0 wt %) and at increasing tannin concentrations (1, 5, 10 wt %). Red arrows indicate the yellow cuvette where 100% of RSA was reached for each sample. (E) Radical scavenging activity (RSA%) as a function of time of the CH-ref and CH-tannin films calculated by using the DPPH^•^ method. (F) Tannin release curves in the water medium as a function of time. Inset shows the final tannin release values (24 h) as a function of the tannin content in the films.

Alongside UV-blocking capacity, the skin’s wound-healing process requires significant protection during the delicate stage of inflammation.^1^ During this stage, various immune cells invade the wound and produce and secrete large amounts of Reactive Oxigen Spieces (ROS), which serve as an essential defense against pathogens. However, if overproduction of ROS occurs, wound-healing complications may arise and lead to chronic conditions.^59^ Therefore, scavenging excess ROS at wound sites could provide an effective treatment to enhance wound repair (Figure 4C). The antioxidant activity of the CH-tannin hydrogels was investigated using the DPPH^•^ method^60^ (detailed procedure in the experimental section), which involves a color transition from purple to yellow when scavenged by an antioxidant. In this study, the DPPH^•^ color transition was shown to occur more rapidly as the tannin content of the hydrogel film increased (Figure 4D). Figure 4E illustrates that the radical scavenging activity (RSA) values, calculated according to eq 5, increased with both tannin content and time. Chitosan itself displayed a moderate scavenging activity, consistent with findings in the literature.^61^ However, when only 1 wt % of tannin was introduced, the rate of scavenging significantly increased, achieving 100% RSA within 5 h. Furthermore, CH-tan5 and CH-tan10 demonstrated comparable and high antioxidant activity, achieving full RSA within the initial 10–20 min of contact with the DPPH^•^ solution. These results indicate that the incorporation of tannins significantly enhanced the antioxidant activity of chitosan-based hydrogels, enabling rapid and complete scavenging of radical species, which could be crucial for accelerating healing and preventing complications during the wound-healing process.

The kinetic release of tannins from the CH-tannin films was also evaluated in water, the main component of wound exudates.^62^ As depicted in Figure 4F, swollen films exhibited rapid tannin release (calculated by eqs 6 and 7) within the initial 4–5 h and then reached a plateau, while the inset reveals that the extent of the release follows an exponential trend with the increase in tannin content. The presence of hydrogen bonding instead of covalent bonding within the CH-tannin hydrogel structure can likely facilitate polyphenol release, suggesting promising release activity of the material during the early stages of wound healing.^63^

To evaluate the biological role of tannins, an initial assessment of the cytotoxicity potential of the CH-tan1 and CH-tan5 films in a culture of murine fibroblasts (L929) was conducted (experimental details are provided in Supporting Information). After 24 h of incubation, CH-tan1 and CH-tan5 films did not exhibit any cytotoxicity effects on the cell culture when compared with untreated cells (*p*-value >0.05, Figure S3A).

Subsequently, further investigation into tannin’s role in wound-healing ability was conducted through two different experimental approaches. First, an *in vitro* scratch test was performed using human fibroblasts, the initial cells involved in the wound-healing process. Then, *ex vivo* tests were conducted using healthy human skin cultures derived from abdominoplasty procedures. During the *in vitro* test, after 24 h of eluate administration (experimental details in Supporting Information), all stimulated fibroblasts exhibited a statistically significant tendency toward increased migration (Figure S3B,C). Specifically, the migratory ability of CH-tan1 and CH-tan5 films reached values of 144 ± 7% and 139 ± 13%, respectively, compared to untreated cells (100%). Similarly, CH-ref films enhanced the migratory rate of treated cells, achieving a value of 133 ± 10%.

In the *ex vivo* tests, human skin organ cultures were topically exposed to 2% SDS to mimic the *in vivo* conditions of barrier perturbation. The ability of tannins to restore skin integrity was evaluated by applying CH-ref, CH-tan1, and CH-tan5 films to damaged skin. Figure 5A displays a scheme of the organ culture experimental setup. Hematoxylin and eosin (H&E) staining revealed that the exposure of human skin organ cultures to SDS strongly affected epidermis morphology, with a visible alteration in stratification (Figure 5B). A misalignment of the stratified cells and a loss of definition between the epidermal layers were observed. Keratinocytes exhibited cellular swelling and cytoplasmic vacuolization, visible as clear spaces within the cells. This effect is caused by the loss of membrane integrity and the influx of fluids, leading to cellular swelling and the formation of vacuoles (Figure 5B). No significant differences in SDS-exposed skin were detected after the topical application of the CH-ref patch, although a slight attempt to recover healthy morphology was noticeable. On the contrary, when the skin was treated with CH-tan1 and CH-tan5 patches, keratinocytes reacquired their physiological architecture, and the skin barrier appeared restored. Specifically, keratinocytes became well-aligned, strongly adhered, and exhibited a defined organization. Additionally, cytoplasmic vacuolization was no longer visible (insets in Figure 5B). Given that SDS exerts its mechanism of action by altering cell–cell junctions and, as a consequence, membrane integrity, immunohistochemistry for claudin 1 expression was performed to highlight the barrier repair effect of chitosan/tannin patches after chemical damage (Figure 5C). In healthy skin, claudin 1 staining revealed a strong, continuous linear staining pattern along the cell borders that resembled a fishing net (in brown), particularly in the upper layers of the epidermis (inset in Figure 5C). The staining uniformity indicates well-maintained cell-to-cell adhesion and a physiologically functional barrier. After SDS incubation, claudin 1 expression was significantly reduced compared with normal skin, reflecting damage to tight junction integrity and a loss of cellular adhesion, contributing to an impaired skin barrier. As expected, the quantification of the claudin 1-specific signal by Image Analysis (Figure 5D) revealed that the levels of the protein in the upper layers of healthy skin were 10.4 ± 3.8-fold higher compared to SDS-treated skin. No significant differences were detected in claudin 1 expression when injured skin was treated with CH-ref. On the contrary, the presence of tannins promoted epidermal regeneration after injury in a dose-dependent manner. In fact, CH-tan1 and CH-tan5 patches increased claudin 1 expression levels by 2.5 ± 0.5 and 4.0 ± 0.4-fold, respectively, compared to untreated damaged skin. Moreover, due to the short half-life of organ cultures,^64^ especially when grown with media that support the keratinocyte component, a calculation model was introduced to predict the required contact time between the patch and the skin to mediate complete damage repair. As expected, the predicted contact times decreased with increasing tannin content in the patch, supporting the potential concentration-dependent role of tannins in healing (inset in Figure 5D). These results highlight tannins as promising additives for enhancing skin wound healing and regeneration, showing their potential ability to restore the skin’s physiological architecture and functional integrity.

**Figure 5:**
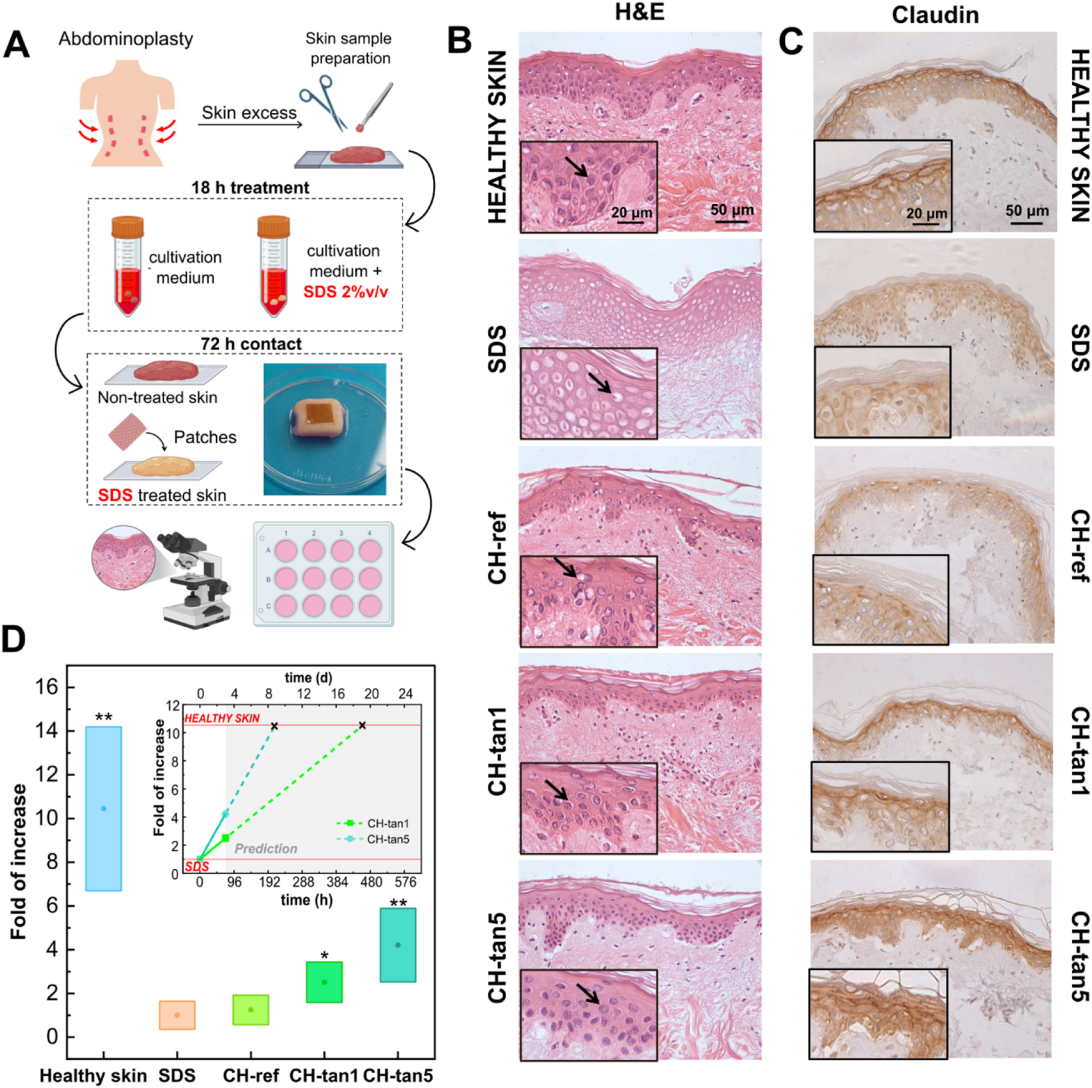
(A) Representative scheme of the organ culture experimental setup. (B) Hematoxylin and eosin (H&E) and (C) claudin 1 expression staining of SDS-perturbed skin following 3 days of contact with CH-ref, CH-tan1, and CH-tan5 patches. Healthy (non-SDS treated) skin and SDS-treated skin without patch application are shown as positive and negative controls, respectively. Black arrows in H&E indicate representative keratinocytes morphology. (D) Claudin 1 expression (at 72 h of incubation) reported as fold increase with respect to SDS sample (8 images for each sample were analyzed). Inset shows time prediction of the fold of increase recovery (at healthy skin levels) of claudin 1 expression for CH-tan1 and CH-tan5. Symbols ** and * represent *p*-value <0.01 and <0.05, respectively. Data shown are representative of 3 independent experiments.

## 3 Conclusions

In this work, fully biobased and multifunctional films for active wound management were developed. The incorporation of wood tannins into chitosan at 1, 5, and 10 wt % allowed the formation of a stable hydrogel with excellent swelling ability (swelling degree >2000%), indicating a high capacity for absorbing wound exudates. Moreover, tensile and adhesion tests demonstrated that improved flexibility and suitable skin adhesion can be obtained after only 5 s-swelling, resulting in a soft material suitable for contact with injured skin and painless dressing changes. The films also showed excellent antioxidant capacity, which is essential for limiting the inflammation stage of the healing process to avoid wound complications. In particular, 100% of RSA was detected through the DPPH radical test for CH-tan1, CH-tan5, and CH-tan10 in 6 h, 30 min, and 10 min, respectively. The increasing tannin content did not affect the transparency of the films, thus allowing continuous monitoring of the wound state, while significantly improving UV-blocking activity, moving from 70% UV absorbance for chitosan to 100% for chitosan containing 5 and 10 wt % tannins. Furthermore, *ex vivo* tests conducted on abdominoplasty-derived human skin showed the effective role of tannins in skin-barrier restoring properties, achieving a 4.4-fold increase in claudin 1 expression compared to insulted skin by applying the CH-tan5 patch for 72 h and enhancing the reacquisition of keratinocytes’ physiological architecture. Overall, the observed antioxidant, anti-inflammatory, and healing properties suggest that the incorporation of tannins into the proposed hydrogel holds potential to enhance wound recovery quality and optimize healing times. These findings open promising therapeutic possibilities for the treatment of wounds, burns, and other skin conditions, with significant potential impact on the field of regenerative medicine.

## 4 Experimental Section

### 4.1 Materials

Chitosan (CH) from shrimp shells (low viscosity, product number 50494), was purchased from Sigma-Aldrich (Milan, Italy) and used as received. Chestnut (Castanea spp.) tannins (kindly supplied by Sadepan Chimica Srl, Italy) were used as received. Formic acid (HCOOH, ≥98%), ethanol (EtOH, ≥99.8%), and 2,2-diphenyl-1-picrylhydrazyl radical (DPPH^•^) were purchased from Sigma-Aldrich (Milan, Italy) and used without further purification.

### 4.2 CH-tannin Film Preparation

The solvent casting method was used to prepare the CH-tannin films. Each CH-tannin film was prepared by dissolving 0.8 g of chitosan in 45 mL of formic acid (2% v/v) and stirring for 24 h at room temperature. Afterward, the solution was filtered using a Gooch crucible (por. 1). Separately, a given amount of tannin, to achieve the desired final concentration (0, 1, 5, or 10 wt %), was weighed and dissolved in 5 mL of formic acid (2% v/v) by stirring for 3 h at room temperature. The filtered chitosan solution was added dropwise to the prepared tannin solution under constant stirring (800 rpm). The final solution was stirred for 4 h at 50 °C, and then, it was left under the hood for overnight degassing. Lastly, the solution was cast into a glass Petri dish (7.5 cm diameter) and dried in an oven at 37 °C. For CH-ref preparation, the same procedure was followed without the addition of tannins. The resulting films had an average thickness of approximately 200 μm. Before further characterization, the films were dried for 16 h at 70 °C under dynamic vacuum conditions.

### 4.3 Fourier Transform Infrared (FT-IR) Spectroscopy

A PerkinElmer Spectrum Two spectrometer equipped with a diamond crystal operating in attenuated total reflectance (ATR) mode was used to analyze the structure of CH-tannin films. Spectra were recorded in the wavenumber region between 4000 and 400 cm^–1^ using a spectral resolution of 4 cm^–1^ and 32 scans for analysis. Spectral data were processed with Spectrum 10 software (PerkinElmer).

### 4.4 Morphological Analysis

Samples were cryogenically fractured under liquid nitrogen to analyze their cross-sectional microstructure. Before analysis, samples were placed onto a copper tape attached to an aluminum stub and metallized with approximately 10 nm of sputtered gold. A Nova NanoSEM 450 electron microscope (FEI Company-Bruker Corporation) was employed, and the analysis was conducted by applying an accelerating voltage of 5 kV.

### 4.5 Swelling Analysis, Gel Content, and Extractable Fraction

Carefully cut specimens with comparable areas of 0.5 × 0.5 cm^2^ were first weighed (*w*_i_) and immersed in 50 mL of distilled water at room temperature for up to 24 h. At different time intervals (5, 10, 15, 20, 25, 30, 45, 60, and 120 s, then at 15, 30, 60, 180, 240 min, and 24 h), the specimens were weighed (*w*_t_). Before each weighing, excess water was wiped from the specimens using filter paper. Considering the low mechanical properties of the long-time swollen hydrogels, a metallic net was used to wrap the specimens (Figure S4). Four specimens were tested for each formulation. After 24 h of swelling, all samples were dried in an oven at 60 °C until a constant weight was reached (*w*_d_). The swelling degree was normalized to the polymer mass and expressed as a swelling percentage, calculated according to the following equation:^65^

1 $$Swelling\;degree\left(\%\cdot mg^{-1}\right)=\left(\left(w_{t}-w_{i}\right)/\left(w_{i}\cdot w_{chitosan}\right)\right)\cdot 100$$

The gel content (the insoluble part of a partially cross-linked polymer specimen) and extractable fractions (the soluble part of a partially cross-linked polymer specimen) were calculated by considering the weight of the samples before water immersion (*w*_i_) and in the completely dry state (*w*_d_):^44^

2 $$Gel\;content\left(\%\right)=\left(w_{d}/w_{i}\right)\cdot 100$$

3 $$Extractable\;fraction\left(\%\right)=\left(w_{i}/w_{d}\right)\cdot 100$$

### 4.6 Water Evaporation

The water loss over time of the 24 h-swollen films was evaluated by using thermogravimetric analysis (TGA, TGA4000, PerkinElmer) operating in isothermal mode at 37 °C. Specifically, pieces of CH-ref and CH-tannin film (0.5 × 0.5 cm^2^) were immersed in distilled water for 24 h. Then, the swollen film was cut to obtain a sample of approximately 15 mg and introduced into a ceramic pan. The test was carried out under an airflow of 20 mL·min^–1^. The sample’s weight loss (%) as a function of time was recorded using Pyris software. The water evaporation rate was calculated as the slope of the weight loss vs time curves in the first 20 min of analysis by using Origin2021 software. All samples were tested in triplicate.

### 4.7 Tensile Test

Tensile properties of the CH-tannin films were assessed using an Instron 5966 device (Instron), equipped with a 10 kN load cell. Specimens in the form of strips (40 × 10 mm^2^) were tested at a crosshead speed of 5 mm·min^–1^. The thickness of each film was calculated as an average from a 3-fold determination. The test was conducted on both dry and swollen films, with increasing swelling times (5, 15, and 30 s). Swelling was achieved through a controlled procedure, in which a 1 cm-wide sponge strip was immersed in distilled water for 5 s and then squeezed under a 0.5 kg weight for 10 s, with excess water removed using blotting paper (Figure S5). The middle part of the film strip was wet for the specified time, and after removing the sponge, a 30 s interval was waited before starting the test to ensure sufficient time for uniform water absorption. The results were reported as mean values and standard deviations from at least five measurements.

### 4.8 Adhesion Test

The skin-adhesion properties of the hydrogels were evaluated by performing a T-peel test (following the guidelines of the ASTM standard test method D1876-08) using a tensile tester, Instron 5567 testing machine (Instron), equipped with a 10 N load cell. First, shaved porcine skin (2.5 × 8 cm^2^) was glued onto a paper support clamped in the lower grip of the tensile tester after the removal of the excess fat layer (analysis setup shown in the inset of Figure 3D). Then, film strips (50 × 15 mm^2^) were immersed for 10 s in water to create the swollen patches (leaving a 10 mm section dry for clamping). The prepared patches were attached to the pig skin and clamped in the upper grip of the machine. The peeling strength was measured at room temperature at a crosshead speed of 10 mm·min^–1^. The average peeling load (N) per specimen width (m) required to separate the adherents was calculated using Instron BlueHill software. A representative video of the test is available in Video SV2. Results were reported as mean values and standard deviations from at least five measurements.

### 4.9 Optical Properties

The optical properties were analyzed using a Jasco V-650 spectrophotometer operating in transmittance mode on specimens measuring 1 × 3 cm^2^. Each spectrum was collected at a scan speed of 400 nm·min^–1^ and a resolution of 0.5 nm within the 200–800 nm range. The reported spectra were obtained by normalization based on the thickness of each film. The transparency of each film was determined from the transmittance values at 600 nm. The UV-blocking activity was calculated as follows:^66^

4 $$UV\text{−}X_{blocking}=100-T_{UV-X}$$

where *T*_UV–X_ is the average transmittance values in the corresponding spectral regions, specifically UV–A (400 to 315 nm) and UV–B (315 to 280 nm). The results were averaged, and standard deviations were calculated based on three measurements for each sample.

### 4.10 Antioxidant Properties

The antioxidant activity of the CH-tannin patches was investigated through the DPPH free radical scavenging assay.^67^ First, a 0.2 mM solution of DPPH^•^ was prepared in a 50:50 ethanol–water mixture and poured into a vial equipped with a magnet. The absorbance spectrum of the DPPH^•^ solution (*A*_0_) was recorded by using a Jasco V-650 spectrophotometer. After the acquisition of the *A*_0_ spectrum, a film sample (0.5 × 1.5 cm^2^) was introduced into the vial, which was kept under stirring in the dark, also ensuring no interference between the magnet and the film. The color change over time of the DPPH^•^ solution was monitored by recording its absorbance spectrum at specific times (1, 5, 10, 20, 30, 60, 90, 120, 180, 240, 300, and 1440 min). This procedure was repeated in triplicate for each film (CH-ref, CH-tan1, CH-tan5, and CH-tan10) and for a reference vial containing DPPH^•^ solution with no sample added. The antioxidant capacity of the films was expressed as RSA%, which was calculated according to the following equation:^66^

5 $$RSA\left(\%\right)=\left(\left(A_{0}-A_{t}\right)/A_{0}\right)\cdot 100$$

where *A*_0_ is the absorbance peak value of DPPH^•^ at *t*_0_ (measured at 515 nm) and *A*_t_ is the absorbance peak value at the specified analysis times.

### 4.11 Tannin Release

The release of tannin from the CH-tannin films was evaluated by UV–visible spectroscopy. Specifically, a specimen of each formulation (dimensions 1 × 3 cm^2^) was initially weighed (*w*_0_) and immersed in a vial containing 8 mL of water at 37 °C. At specific time intervals, 4 mL was transferred from the vial to a cuvette and analyzed by a Jasco V-650 spectrophotometer operating in absorbance mode. After the measurement, the volume contained in the cuvette was reintroduced into the vial. This process was repeated at time intervals of 1, 5, 10, 20, 30, 60, 90, 120, 180, 240, and 300 min. The tannin release kinetic was monitored by the variation of the absorbance peak at 280 nm, which is characteristic of the phenolic structure of tannins.^68^ The tannin concentration (*C*_t_) at each time point was determined using a previously prepared calibration curve (absorbance *vs* tannin concentration), and the total amount of released tannin was then reported in mg (*w*_t_).

6 $$w_{t}\left(mg\right)=C_{t}\cdot V$$

where *V* is the volume of water in the vial (8 mL). The tannin release was expressed as the mass of tannin released per superficial area of the analyzed sample and was calculated as follows:

7 $$\mathrm{Tan}nin\;release\left(mg\cdot cm^{-2}\right)=w_{t}/A_{sup}$$

where *A*_sup_ is the superficial area of the tested specimen (considering both faces of the film).

### 4.12 Skin Organ Culture

Organ cultures were obtained from healthy adult female volunteers (*n* = 3) who underwent abdominoplasty surgery for esthetic purposes. Enrolled patients provided informed written consent for the use of skin biopsies derived from their tissue. The use of human tissue specimens was approved by the Ethical Committee of Modena (Protocol No. AOU 0033466/19, Comitato Etico dell’Area Vasta Emilia Nord, December 4, 2019). The skin barrier was perturbed by incubation with 2% SDS (Sigma-Aldrich, St Louis, MO, USA) at 4 °C for 18 h. Tannin-based patches (CH-ref, CH-tan1, and CH-tan5) were applied on top of SDS-treated skin, in direct contact with the epidermis, and cultured in an air liquid interface for 72 h in DMEM (Life Technologies, Carlsbad, CA, USA) enriched with 10% fetal bovine serum, 1% penicillin/streptomycin (Corning, Billings, MT, USA), and 2% glutamine (Corning, Billings, MT, USA). Skin organ cultures not receiving tannin-enriched patches were considered negative controls for the treatment, while healthy skin not incubated with SDS was considered as negative control for evaluating the activity of SDS in barrier disruption.

### 4.13 Hematoxylin and Eosin

Skin biopsies were fixed in 4% neutral-buffered formalin (Histo-Line Laboratories, MI, Italy) in PBS for 24 h. After dehydration in an ascending alcohol series (Histo-Line Laboratories), samples were embedded in paraffin. Four μm thick sections were stained with hematoxylin and eosin (Diapath, Martinengo, BG, Italy) according to the manufacturer’s instructions. Briefly, samples were sliced into 4 μm thick sections, dyed by hematoxylin and eosin staining, and mounted in DPX (Sigma-Aldrich). Images were acquired by AxioZoom M2 (Zeiss, Oberkochen, Germany) at a magnification of 40× (objective EC-PLAN-NEOFLUAR 40*X*/0.75 420360-9900) and analyzed by ZEN PRO software (Zeiss).

### 4.14 Immunohistochemistry

Formalin-fixed, paraffin-embedded skin specimens were cut and stained to detect claudin 1 expression. Sections were retrieved in citrate buffer (Carlo Erba, Arese, MI, Italy) for 15 min at 96 °C and 20 min at room temperature and incubated overnight at 4 °C with the primary antirabbit claudin 1 antibody at a dilution of 1:200 (ab140349, Abcam, Cambridge, UK) to mark tight junctions. Slides were then incubated with a biotinylated goat antirabbit IgG (H + L) at a dilution of 1:200 (Vector Laboratories, Burlingame, CA; #BA9200) for 1 h at room temperature. Negative controls were run simultaneously, omitting the primary antibody while incubating with buffer. Staining was performed and visualized in brown by 3,3’-diaminobenzidine (DAB, Vector Laboratories; #SK-4100). All slides were counterstained with hematoxylin (Diapath). Images were acquired by AxioZoom M2 (Zeiss, Oberkochen, Germany) at magnifications of 20× and 40× (objective) and analyzed by ZEN PRO software (Zeiss). Quantification of the area stained by the antibody was performed by ImageAnalysis plugin (ZEN Pro, Zeiss; 8 images for each sample were analyzed). The positive area was expressed as the fold increase and normalized to the SDS-treated sample. The Student *t*-test was performed to compare data between samples and the SDS-treated skin. *p*<0.05 (*) and *p*<0.01 (**) were considered statistically significant.

## Supplementary Material







## Notes

**Video SV1:** A proof-of-concept application of the CH-tan5 patch adhered to finger skin during joint movement (MP4)
**Video SV2**: A representative T-peel test used to assess adhesion strength (MP4)
Mechanical properties of hydrogels as a function of the swelling time, scheme of the adhesion experiment setup; experimental details and results of cell culture and wound healing assay; representative images of swelling test sample holders and mechanical tensile tests (PDF)
