# Supplementary material for: Skin Barrier Restoration by Waste-Derived Multifunctional Adhesive Hydrogel Based on Tannin-Modified Chitosan
Source: ACS Appl Mater Interfaces. 2025 May 19;17(24):35066–79. doi: 10.1021/acsami.5c03066 (PMC12186222; doi:10.1021/acsami.5c03066)
Supplement: Supplementary file 3 [file am5c03066_si_003.pdf]

SUPPORTING INFORMATION

Skin barrier restoration by waste-derived multifunctional adhesive hydrogel based on tannin-modified chitosan

Martina Ferri [a,b], Francesco Ganzerli [c], Alberto Portone [c], Tiziana Petrachi [c], Elena Veronesi [c], Davide Morselli [a,b*], Micaela Degli Esposti [a,b*], and Paola Fabbri [a,b]

[a] *Department of Civil, Chemical, Environmental and Materials Engineering (DICAM), Università di Bologna, Via Terracini 28, 40131, Bologna, Italy*

[b] *National Interuniversity Consortium of Materials Science and Technology (INSTM), Via Giusti 9, 50121, Firenze, Italy*

[c] *Tecnopolo Mario Veronesi, Via 29 Maggio 6, 41037, Mirandola, Modena, Italy*

* Corresponding Authors: Micaela Degli Esposti (micaela.degliesposti@unibo.it); Davide Morselli (davide.morselli6@unibo.it)

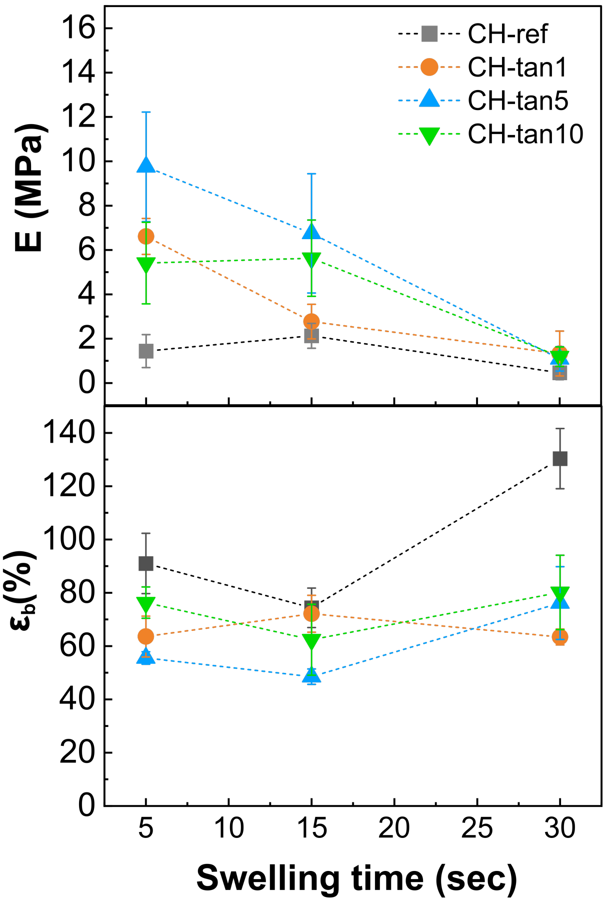

Figure S1. Young's modulus (E) and elongation at break (ε_b) of the hydrogel films as a function of the swelling time.

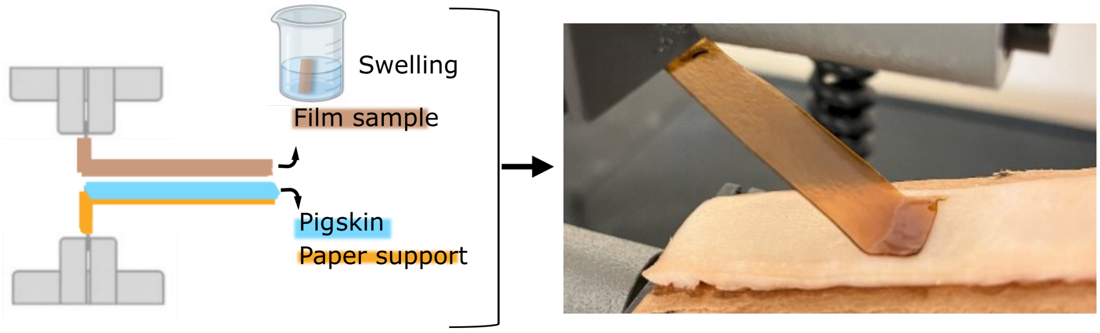

Figure S2. Scheme of the adhesion experiment setup.

Cell culture. L929 murine fibroblasts (Sigma-Aldrich, St. Louis, MO, USA) were seeded according to the manufacturer's instructions at a density of 15,000 cells/cm² in T75 flasks, using Dulbecco's Modified Eagle's Medium (DMEM; Gibco, Bleiswijk, the Netherlands) containing 15% fetal bovine serum (FBS; Euroclone, Pero, Italy), 1% penicillin/streptomycin (Gibco, Grand Island, NY, USA), and 2% glutamine (Gibco, Grand Island, NY, USA), and maintained at 37 °C in a 5% CO_2 atmosphere. Five days after seeding, cells reaching 90% confluence were detached using 0.05% trypsin-EDTA (0.02%; Gibco, Paisley, UK), counted using 0.4% trypan blue (Sigma-Aldrich, Gillingham, UK) for viability exclusion testing, seeded into 96-well plates (10,000 cells/100 µL/well), and maintained in culture. After 24 h, cells were stimulated with the obtained extract. Extracts were filtered through a 0.22 µm filter and diluted to a concentration of 10%. Latex (Adventa Health, Kota Bharu, Malaysia) and high density polyethylene (HDPE; Sigma-Aldrich, St. Louis, MO, USA) were used as positive and negative controls, respectively.

After 24 h of exposure, cells were incubated with 50 µL of MTT solution (Sigma-Aldrich, St. Louis, MO, USA) for 2 h at 37 °C. Cell viability was assessed by measuring the optical density at 570 nm using a multi-well plate reader spectrophotometer (Enspire, PerkinElmer, Hopkinton, MA, USA) after removing the MTT solution and suspending the cells in 100 µL of isopropanol (Sigma-Aldrich, St. Louis, MO, USA). The reduction in cell viability compared to the negative control was determined using the following equation:

$$Cell\ viability\ (\%) = (OD570_e / OD570\_b) \times 100 \qquad \textit{Equation S1}$$

where $OD570_e$ is the mean optical density of the 10% extract, and *OD570_b* is the mean optical density of the blanks. A viability greater than 70% of the blank was considered non-cytotoxic. Each experiment was performed in sextuplicate. Statistical analysis was conducted using the Student *t*-test to compare treated samples with untreated cells. $p < 0.05$ (*), $p < 0.01$ (**), $p < 0.001$ (***) were considered statistically significant.

Wound healing assay. The impact of CH-tan1 and CH-tan5 films on the migration of primary skin fibroblast cells (HFFs; ATCC, Manassas, VE, USA) was evaluated by wound healing scratch test. Cells were seeded at the density of 200000 cells in 12-well plates in DMEM supplemented with 15% fetal bovine serum (FBS; Corning, Woodland, CA), 2% L-glutamine 200 mM (Gibco) and 1% penicillin streptomycin (Gibco). Once confluence was reached, a pipette tip (1000 μL) was used to eliminate the cells adhering to the middle of the well. Wells

were washed with PBS twice. Cells were then treated with extracts obtained from CH-ref, CH-tan1, and CH-tan5 films extracts for 24 h. After 24 h, cells were fixed with 4% formaldehyde and stained with Cristal Violet (0.4%, Sigma Aldrich) for 20 min at room temperature. Migrated cells were observed by Axio Observer.Z1 (Zeiss), counted from three different areas. Results were expressed as the mean ± standard deviation and reported to negative control (100%). The Student *t*-test was performed to compare data between samples and the untreated cells (control). $p < 0.05$ (*), $p < 0.01$ (**), $p < 0.001$ (***) were considered statistically significant.

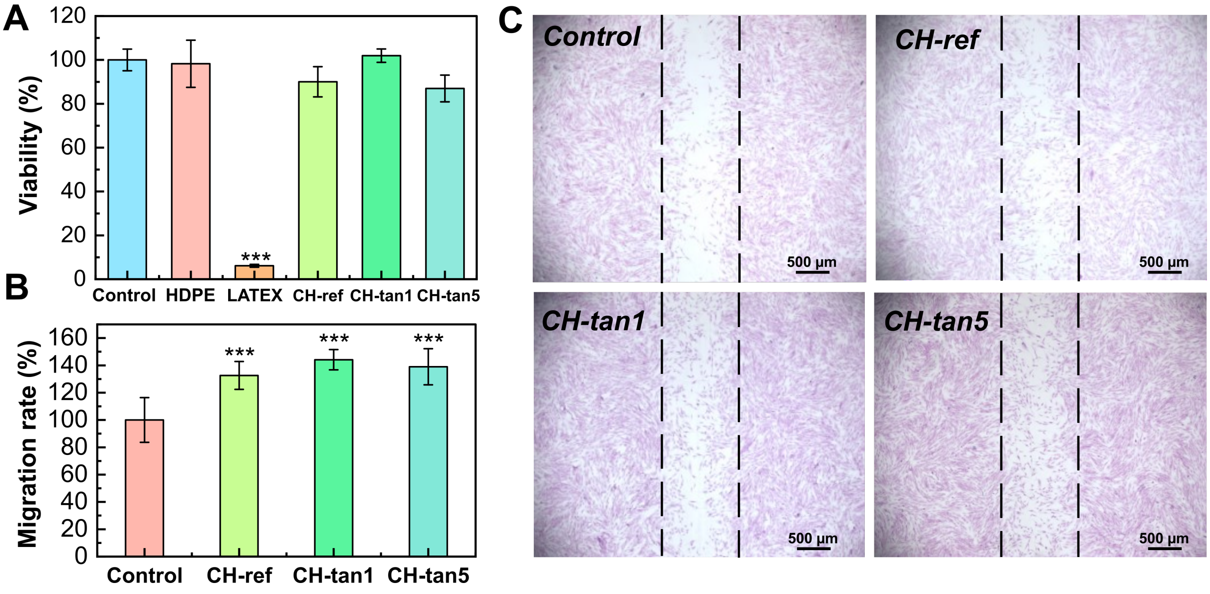

Figure S3. (A) Percentage of cell viability of L929 murine fibroblasts after treatment with extracts from CH-ref, CH-tan and CH-tan5. Control refers to untreated cells. HDPE and Latex were used as negative and positive control, respectively. Each experiment was performed in sextuplicate. The Student *t*-test was performed to compare data between samples and the untreated cells (control; *** indicate a *p*-value < 0.001). **(B)** Percentage of cell migration after 24 hours of treatment with extracts from CH-ref, CH-tan and CH-tan5. Three different areas per group were counted. The Student *t*-test was performed to compare data between samples and the untreated cells (control; *** indicate a *p*-value < 0.001). **(C)** Representative photomicrograph of wound edge in scratch assay.

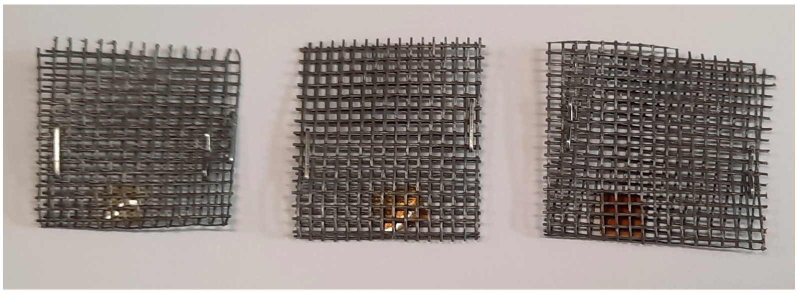

Figure S4. Metallic wrapping nets used for the swelling tests.

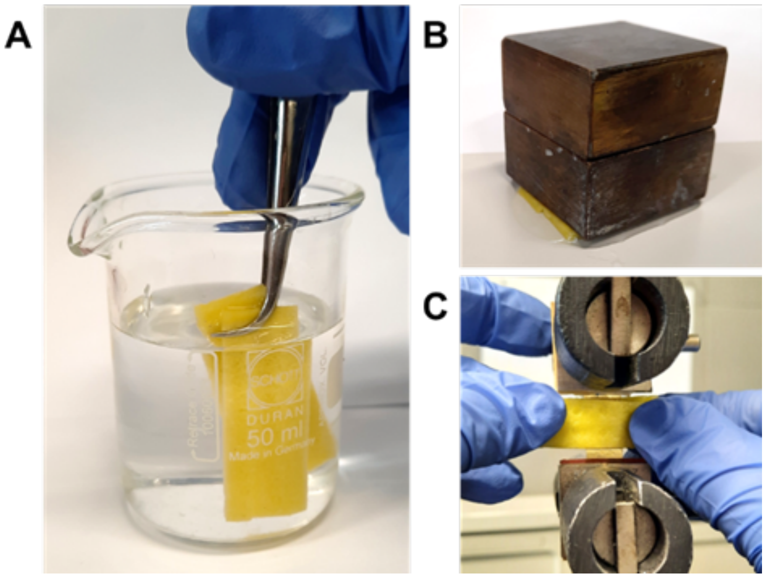

Figure S5. Three-steps procedure to obtain swelled samples with an equal degree of moisturizing before the tensile test. **(A)** Dipping in distilled water of 1 cm-width sponge stripes. **(B)** Removing water excess from the sponge stripes, using 2 weights of 250 g each. **(C)** Application of the sponge stripes on the CH-tannin films before starting the tensile test to achieve the desired moisturizing environment.
